# 1-(4-Amino­phen­yl)-3-[2-(trifluoro­meth­yl)phen­yl]prop-2-en-1-one

**DOI:** 10.1107/S1600536810014169

**Published:** 2010-04-24

**Authors:** Jing Peng, Huifen Xu, Zhe Li, Yuyan Zhang, Jianzhang Wu

**Affiliations:** a School of Pharmacy, Wenzhou Medical College, Wenzhou, Zhejiang Province 325035, People’s Republic of China; b Life Science College, Wenzhou Medical College, Wenzhou, Zhejiang Province 325035, People’s Republic of China; c Institute of Biotechnology, Nanjing University of Science and Technology, Nanjing, Jiangsu Province 210094, People’s Republic of China

## Abstract

The title compound, C_16_H_12_F_3_NO, a derivative of biologically active chalcones, comprises two benzene rings and a central –CH=CH—C(=O)– unit. The dihedral angle between the two rings is 10.9 (1)° and the mol­ecule adopts an *E* configuration about the central olefinic bond. The crystal structure is stabilized by inter­molecular N—H⋯O and N—H⋯N hydrogen bonds.

## Related literature

For related structures, see: Narender *et al.* (2007 ▶); Kamal *et al.* (2008 ▶); Wu *et al.* (2009 ▶); Low *et al.* (2002 ▶); Yathirajan *et al.* (2006 ▶); Suwunwong *et al.* (2009 ▶). For background to and applications of chalcones, see: Heidari *et al.* (2009 ▶); Nielsen *et al.* (2005 ▶); Mojzis *et al.* (2008 ▶); Achanta *et al.* (2006 ▶); Dimmock *et al.* (1999 ▶); Liang *et al.* (2007*a* ▶,*b* ▶, 2009 ▶); Zhao *et al.* (2010 ▶).
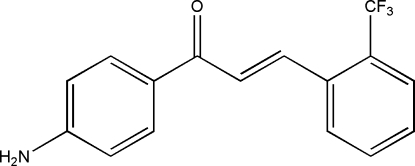


## Experimental

### 

#### Crystal data

C_16_H_12_F_3_NO
*M* *_r_* = 291.27
Monoclinic, 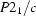

*a* = 18.835 (3) Å
*b* = 4.7866 (8) Å
*c* = 15.177 (3) Å
β = 101.108 (3)°
*V* = 1342.7 (4) Å^3^
*Z* = 4
Mo *K*α radiation
μ = 0.12 mm^−1^
*T* = 273 K
0.43 × 0.28 × 0.22 mm

#### Data collection

Bruker APEXII CCD area-detector diffractometer
Absorption correction: multi-scan (*SADABS*; Bruker, 2001 ▶) *T*_min_ = 0.951, *T*_max_ = 0.974
6607 measured reflections
2360 independent reflections
1700 reflections with *I* > 2σ(*I*)
*R*_int_ = 0.130

#### Refinement

*R*[*F* ^2^ > 2σ(*F* ^2^)] = 0.060
*wR*(*F* ^2^) = 0.185
*S* = 1.00
2360 reflections
191 parameters
H-atom parameters constrained
Δρ_max_ = 0.23 e Å^−3^
Δρ_min_ = −0.25 e Å^−3^

### 

Data collection: *APEX2* (Bruker, 2004 ▶); cell refinement: *SAINT-Plus* (Bruker, 2001 ▶); data reduction: *SAINT-Plus*; program(s) used to solve structure: *SHELXS97* (Sheldrick, 2008 ▶); program(s) used to refine structure: *SHELXL97* (Sheldrick, 2008 ▶); molecular graphics: *SHELXTL* (Sheldrick, 2008 ▶); software used to prepare material for publication: *SHELXTL*.

## Supplementary Material

Crystal structure: contains datablocks I, global. DOI: 10.1107/S1600536810014169/zq2037sup1.cif

Structure factors: contains datablocks I. DOI: 10.1107/S1600536810014169/zq2037Isup2.hkl

Additional supplementary materials:  crystallographic information; 3D view; checkCIF report

## Figures and Tables

**Table 1:** Hydrogen-bond geometry (Å, °)

*D*—H⋯*A*	*D*—H	H⋯*A*	*D*⋯*A*	*D*—H⋯*A*
N1—H1*A*⋯N1^i^	0.86	2.42	3.235 (3)	158
N1—H1*B*⋯O1^ii^	0.86	2.45	3.162 (3)	140

Symmetry codes: (i) 
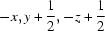
; (ii) 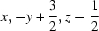.

**Fig. 1.**
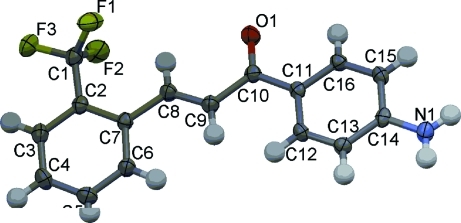
The molecular structure of the title compound, showing 30% displacement ellipsoids for the non-hydrogen atoms. Hydrogen atoms are drawn as spheres of arbitrary radius.

## supplementary crystallographic information

### Comment

Chalcones, which are open-chain flavonoids, distribute widespread in fruits, vegetables and so on. Chalcone and their derivatives are obtained from the aldol condensation of aromatic aldehydes and aromatic ketones. They are important intermediate of organic synthesis. Due to their significant biological activities such as anti-inflammatory, antibacterial, antiangiogenic, antitumor and analgesic, they have attracted more and more attention (Heidari *et al.*, 2009; Nielsen *et al.*, 2005; Mojzis *et al.*, 2008; Achanta *et al.*, 2006; Dimmock *et al.*, 1999). The molecule of chalcone possess two phenyl rings and one –CH=CH–C(=O)– central part. The carbonyl functional group which is responsible for the antibacterial activity of these compounds is the main feature of chalcone derivatives (Suwunwong *et al.*, 2009).

Due to the broad spectrum of biological activities of this type of compounds, various chalcone analogues have been synthesized in order to filter the better ones or the unique ones (Narender *et al.*, 2007; Kamal *et al.*, 2008). As a continuation of our broad program of work on the synthesis and structural study of chalcones, the title chalcone derivative has been obtained and an X-ray diffraction study was carried out.

The molecule is approximately planar and the dihedral angle between the two phenyl rings is 10.9 (1)°. The H atoms of the central propenone group are *trans *to each other. The average value of the phenyl bond distances [1.385 (5) Å] and bond angles [120.7 (4)°] have normal values which agree quite well with the values reported in the literature for some analogous structures (Wu *et al.*, 2009; Low *et al.*, 2002; Yathirajan *et al.*, 2006). The crystal structure is stabilized by intermolecular N(1)–H1A···O(1) and N(1)–H1B···N(1) hydrogen bonds.

### Experimental

1-(4-aminophenyl)ethanone (5 mmol) was dissolved in ethanol (10 ml) and a solution of KOH (40%, 5 drops) was added. The flask was immersed in bath of crushed ice and a solution of 2-(trifluoromethyl)benzaldehyde (5 mmol) in ethanol (10 ml) was added. The reaction mixture was stirred at 300 K and completion of the reaction was monitored by thin-layer chromatography. Ice-cold water was added to the reaction mixture after 24 h and the yellow solid that separated was filtered off, washed with water and cold ethanol, dried and purified by column chromatography on silica gel (yield: 68%). Single crystals of the title compound were grown in a CH_2_Cl_2_/CH_3_OH mixture (7:3 *v*/*v*) by slow evaporation at 277 K.

### Refinement

The H atoms were positioned geometrically (C—H = 0.93 and N—H = 0.86 Å) and refined as riding with *U*_iso_(H) = 1.2*U*_eq_(C,N).

### Crystal data

**Table:**

C_16_H_12_F_3_NO	*F*(000) = 600
*M**_r_* = 291.27	*D*_x_ = 1.441 Mg m^−^^3^
Monoclinic, *P*2_1_/*c*	Mo *K*α radiation, λ = 0.71073 Å
Hall symbol: -P 2ybc	Cell parameters from 1903 reflections
*a* = 18.835 (3) Å	θ = 2.7–25.4°
*b* = 4.7866 (8) Å	µ = 0.12 mm^−^^1^
*c* = 15.177 (3) Å	*T* = 273 K
β = 101.108 (3)°	Block, colorless
*V* = 1342.7 (4) Å^3^	0.43 × 0.28 × 0.22 mm
*Z* = 4	

### Data collection

**Table:**

Bruker APEXII CCD area-detector diffractometer	2360 independent reflections
Radiation source: fine-focus sealed tube	1700 reflections with *I* > 2σ(*I*)
graphite	*R*_int_ = 0.130
φ and ω scans	θ_max_ = 25.0°, θ_min_ = 1.1°
Absorption correction: multi-scan (*SADABS*; Bruker, 2001)	*h* = −21→22
*T*_min_ = 0.951, *T*_max_ = 0.974	*k* = −5→5
6607 measured reflections	*l* = −18→10

### Refinement

**Table:**

Refinement on *F*^2^	Secondary atom site location: difference Fourier map
Least-squares matrix: full	Hydrogen site location: inferred from neighbouring sites
*R*[*F*^2^ > 2σ(*F*^2^)] = 0.060	H-atom parameters constrained
*wR*(*F*^2^) = 0.185	*w* = 1/[σ^2^(*F*_o_^2^) + (0.1025*P*)^2^] where *P* = (*F*_o_^2^ + 2*F*_c_^2^)/3
*S* = 1.00	(Δ/σ)_max_ = 0.001
2360 reflections	Δρ_max_ = 0.23 e Å^−^^3^
191 parameters	Δρ_min_ = −0.25 e Å^−^^3^
0 restraints	Extinction correction: *SHELXL97* (Sheldrick, 2008), Fc^*^=kFc[1+0.001xFc^2^λ^3^/sin(2θ)]^-1/4^
Primary atom site location: structure-invariant direct methods	Extinction coefficient: 0.005 (3)

### Special details

**Table:**

Geometry. All esds (except the esd in the dihedral angle between two l.s. planes) are estimated using the full covariance matrix. The cell esds are taken into account individually in the estimation of esds in distances, angles and torsion angles; correlations between esds in cell parameters are only used when they are defined by crystal symmetry. An approximate (isotropic) treatment of cell esds is used for estimating esds involving l.s. planes.
Refinement. Refinement of *F*^2^ against ALL reflections. The weighted *R*-factor wR and goodness of fit *S* are based on *F*^2^, conventional *R*-factors *R* are based on *F*, with *F* set to zero for negative *F*^2^. The threshold expression of *F*^2^ > σ(*F*^2^) is used only for calculating *R*-factors(gt) etc. and is not relevant to the choice of reflections for refinement. *R*-factors based on *F*^2^ are statistically about twice as large as those based on *F*, and *R*- factors based on ALL data will be even larger.

### Fractional atomic coordinates and isotropic or equivalent isotropic displacement parameters (Å^2^)

**Table:**

	*x*	*y*	*z*	*U*_iso_*/*U*_eq_	
C1	0.34496 (14)	−0.3803 (5)	0.79335 (18)	0.0501 (6)	
C2	0.36516 (11)	−0.4190 (5)	0.70442 (16)	0.0410 (6)	
C3	0.42148 (13)	−0.6043 (6)	0.69850 (19)	0.0522 (7)	
H3	0.4457	−0.6952	0.7497	0.063*	
C4	0.44121 (14)	−0.6525 (6)	0.6172 (2)	0.0604 (8)	
H4	0.4786	−0.7762	0.6135	0.073*	
C5	0.40580 (14)	−0.5182 (6)	0.5413 (2)	0.0593 (8)	
H5	0.4193	−0.5502	0.4864	0.071*	
C6	0.34998 (13)	−0.3352 (6)	0.54698 (18)	0.0529 (7)	
H6	0.3263	−0.2464	0.4950	0.063*	
C7	0.32798 (12)	−0.2792 (5)	0.62745 (16)	0.0412 (6)	
C8	0.26836 (13)	−0.0800 (5)	0.62980 (17)	0.0476 (6)	
H8	0.2543	−0.0556	0.6848	0.057*	
C9	0.23372 (13)	0.0641 (5)	0.56274 (18)	0.0486 (6)	
H9	0.2468	0.0408	0.5072	0.058*	
C10	0.17464 (12)	0.2634 (5)	0.56880 (16)	0.0416 (6)	
C11	0.14526 (11)	0.4364 (4)	0.48894 (16)	0.0375 (6)	
C12	0.17160 (12)	0.4283 (5)	0.40932 (17)	0.0449 (6)	
H12	0.2090	0.3057	0.4046	0.054*	
C13	0.14390 (12)	0.5965 (5)	0.33733 (17)	0.0469 (6)	
H13	0.1628	0.5861	0.2852	0.056*	
C14	0.08774 (11)	0.7824 (5)	0.34190 (16)	0.0416 (6)	
C15	0.05970 (12)	0.7888 (5)	0.42057 (18)	0.0459 (6)	
H15	0.0215	0.9084	0.4246	0.055*	
C16	0.08779 (12)	0.6204 (5)	0.49211 (17)	0.0431 (6)	
H16	0.0683	0.6286	0.5439	0.052*	
F1	0.34613 (11)	−0.1107 (3)	0.81944 (11)	0.0793 (6)	
F2	0.27876 (9)	−0.4720 (4)	0.79671 (12)	0.0768 (6)	
F3	0.38918 (9)	−0.5133 (4)	0.86064 (11)	0.0736 (6)	
N1	0.05877 (11)	0.9453 (4)	0.26847 (15)	0.0543 (6)	
H1A	0.0227	1.0535	0.2707	0.065*	
H1B	0.0768	0.9384	0.2206	0.065*	
O1	0.15200 (10)	0.2839 (4)	0.63840 (13)	0.0660 (6)	

### Atomic displacement parameters (Å^2^)

**Table:**

	*U*^11^	*U*^22^	*U*^33^	*U*^12^	*U*^13^	*U*^23^
C1	0.0540 (15)	0.0454 (14)	0.0489 (15)	0.0024 (11)	0.0046 (12)	0.0064 (12)
C2	0.0363 (12)	0.0409 (12)	0.0441 (13)	−0.0044 (10)	0.0037 (10)	0.0006 (11)
C3	0.0437 (14)	0.0539 (14)	0.0552 (16)	0.0059 (11)	0.0000 (12)	0.0051 (13)
C4	0.0424 (14)	0.0695 (17)	0.0703 (19)	0.0150 (13)	0.0132 (13)	−0.0025 (15)
C5	0.0568 (16)	0.0717 (18)	0.0522 (16)	0.0089 (14)	0.0179 (14)	−0.0028 (14)
C6	0.0482 (14)	0.0662 (16)	0.0442 (14)	0.0107 (13)	0.0087 (12)	0.0049 (13)
C7	0.0352 (12)	0.0448 (13)	0.0432 (13)	−0.0002 (10)	0.0065 (10)	0.0021 (11)
C8	0.0443 (13)	0.0562 (14)	0.0439 (14)	0.0075 (11)	0.0127 (11)	0.0048 (12)
C9	0.0473 (14)	0.0549 (15)	0.0441 (14)	0.0092 (11)	0.0100 (12)	0.0055 (12)
C10	0.0351 (12)	0.0461 (13)	0.0433 (14)	−0.0020 (10)	0.0070 (10)	−0.0018 (11)
C11	0.0289 (11)	0.0391 (12)	0.0443 (13)	−0.0040 (9)	0.0063 (10)	−0.0018 (10)
C12	0.0340 (12)	0.0507 (14)	0.0508 (14)	0.0063 (10)	0.0103 (11)	−0.0009 (12)
C13	0.0407 (13)	0.0590 (15)	0.0430 (14)	0.0018 (11)	0.0130 (11)	0.0022 (12)
C14	0.0314 (11)	0.0433 (12)	0.0469 (14)	−0.0070 (9)	−0.0009 (10)	0.0021 (11)
C15	0.0352 (12)	0.0487 (13)	0.0542 (15)	0.0075 (10)	0.0093 (11)	0.0002 (12)
C16	0.0375 (12)	0.0496 (13)	0.0438 (13)	−0.0004 (10)	0.0118 (10)	−0.0019 (11)
F1	0.1293 (15)	0.0565 (10)	0.0519 (10)	0.0041 (10)	0.0173 (10)	−0.0057 (8)
F2	0.0622 (10)	0.1068 (14)	0.0678 (11)	−0.0078 (9)	0.0287 (9)	0.0063 (10)
F3	0.0831 (12)	0.0847 (12)	0.0489 (10)	0.0169 (9)	0.0029 (9)	0.0161 (8)
N1	0.0472 (12)	0.0637 (13)	0.0503 (13)	0.0050 (10)	0.0052 (10)	0.0114 (11)
O1	0.0664 (12)	0.0844 (14)	0.0514 (11)	0.0276 (10)	0.0223 (10)	0.0148 (10)

### Geometric parameters (Å, °)

**Table:**

C1—F1	1.349 (3)	C9—C10	1.482 (3)
C1—F2	1.332 (3)	C9—H9	0.9300
C1—F3	1.347 (3)	C10—O1	1.217 (3)
C1—C2	1.483 (4)	C10—C11	1.484 (3)
C2—C3	1.399 (3)	C11—C16	1.404 (3)
C2—C7	1.410 (3)	C11—C12	1.392 (3)
C3—C4	1.375 (4)	C12—C13	1.376 (3)
C3—H3	0.9300	C12—H12	0.9300
C4—C5	1.374 (4)	C13—C14	1.394 (3)
C4—H4	0.9300	C13—H13	0.9300
C5—C6	1.384 (4)	C14—N1	1.383 (3)
C5—H5	0.9300	C14—C15	1.396 (3)
C6—C7	1.389 (3)	C15—C16	1.374 (3)
C6—H6	0.9300	C15—H15	0.9300
C7—C8	1.479 (3)	C16—H16	0.9300
C8—C9	1.296 (4)	N1—H1A	0.8600
C8—H8	0.9300	N1—H1B	0.8600
			
F1—C1—F2	105.4 (2)	C8—C9—C10	124.3 (2)
F1—C1—F3	104.9 (2)	C8—C9—H9	117.8
F2—C1—F3	105.2 (2)	C10—C9—H9	117.8
F1—C1—C2	113.2 (2)	O1—C10—C11	121.7 (2)
F2—C1—C2	113.6 (2)	O1—C10—C9	119.9 (2)
F3—C1—C2	113.7 (2)	C11—C10—C9	118.3 (2)
C3—C2—C7	120.6 (2)	C16—C11—C12	116.9 (2)
C3—C2—C1	117.9 (2)	C16—C11—C10	119.4 (2)
C7—C2—C1	121.4 (2)	C12—C11—C10	123.7 (2)
C4—C3—C2	120.2 (3)	C13—C12—C11	121.9 (2)
C4—C3—H3	119.9	C13—C12—H12	119.0
C2—C3—H3	119.9	C11—C12—H12	119.0
C3—C4—C5	120.1 (2)	C12—C13—C14	120.6 (2)
C3—C4—H4	119.9	C12—C13—H13	119.7
C5—C4—H4	119.9	C14—C13—H13	119.7
C6—C5—C4	119.7 (3)	N1—C14—C15	121.4 (2)
C6—C5—H5	120.1	N1—C14—C13	120.3 (2)
C4—C5—H5	120.1	C15—C14—C13	118.2 (2)
C5—C6—C7	122.4 (3)	C16—C15—C14	120.8 (2)
C5—C6—H6	118.8	C16—C15—H15	119.6
C7—C6—H6	118.8	C14—C15—H15	119.6
C6—C7—C2	116.9 (2)	C15—C16—C11	121.6 (2)
C6—C7—C8	120.1 (2)	C15—C16—H16	119.2
C2—C7—C8	123.0 (2)	C11—C16—H16	119.2
C9—C8—C7	126.4 (2)	C14—N1—H1A	120.0
C9—C8—H8	116.8	C14—N1—H1B	120.0
C7—C8—H8	116.8	H1A—N1—H1B	120.0

### Hydrogen-bond geometry (Å, °)

**Table:**

*D*—H···*A*	*D*—H	H···*A*	*D*···*A*	*D*—H···*A*
N1—H1A···N1^i^	0.86	2.42	3.235 (3)	158
N1—H1B···O1^ii^	0.86	2.45	3.162 (3)	140

Symmetry codes: (i) −*x*, *y*+1/2, −*z*+1/2; (ii) *x*, −*y*+3/2, *z*−1/2.
